# Positive or negative environmental modulations on human brain development: the morpho-functional outcomes of music training or stress

**DOI:** 10.3389/fnins.2023.1266766

**Published:** 2023-11-03

**Authors:** Carla Mucignat-Caretta, Giulia Soravia

**Affiliations:** ^1^Department of Molecular Medicine, University of Padova, Padova, Italy; ^2^Department of Mother and Child Health, University of Padova, Padova, Italy

**Keywords:** development, brain, behavior, childhood, environment, music, stress

## Abstract

In the last couple of decades, the study of human living brain has benefitted of neuroimaging and non-invasive electrophysiological techniques, which are particularly valuable during development. A number of studies allowed to trace the usual stages leading from pregnancy to adult age, and relate them to functional and behavioral measurements. It was also possible to explore the effects of some interventions, behavioral or not, showing that the commonly followed pathway to adulthood may be steered by external interventions. These events may result in behavioral modifications but also in structural changes, in some cases limiting plasticity or extending/modifying critical periods. In this review, we outline the healthy human brain development in the absence of major issues or diseases. Then, the effects of negative (different stressors) and positive (music training) environmental stimuli on brain and behavioral development is depicted. Hence, it may be concluded that the typical development follows a course strictly dependent from environmental inputs, and that external intervention can be designed to positively counteract negative influences, particularly at young ages. We also focus on the social aspect of development, which starts *in utero* and continues after birth by building social relationships. This poses a great responsibility in handling children education and healthcare politics, pointing to social accountability for the responsible development of each child.

## 1. Introduction

**Figure 1:**
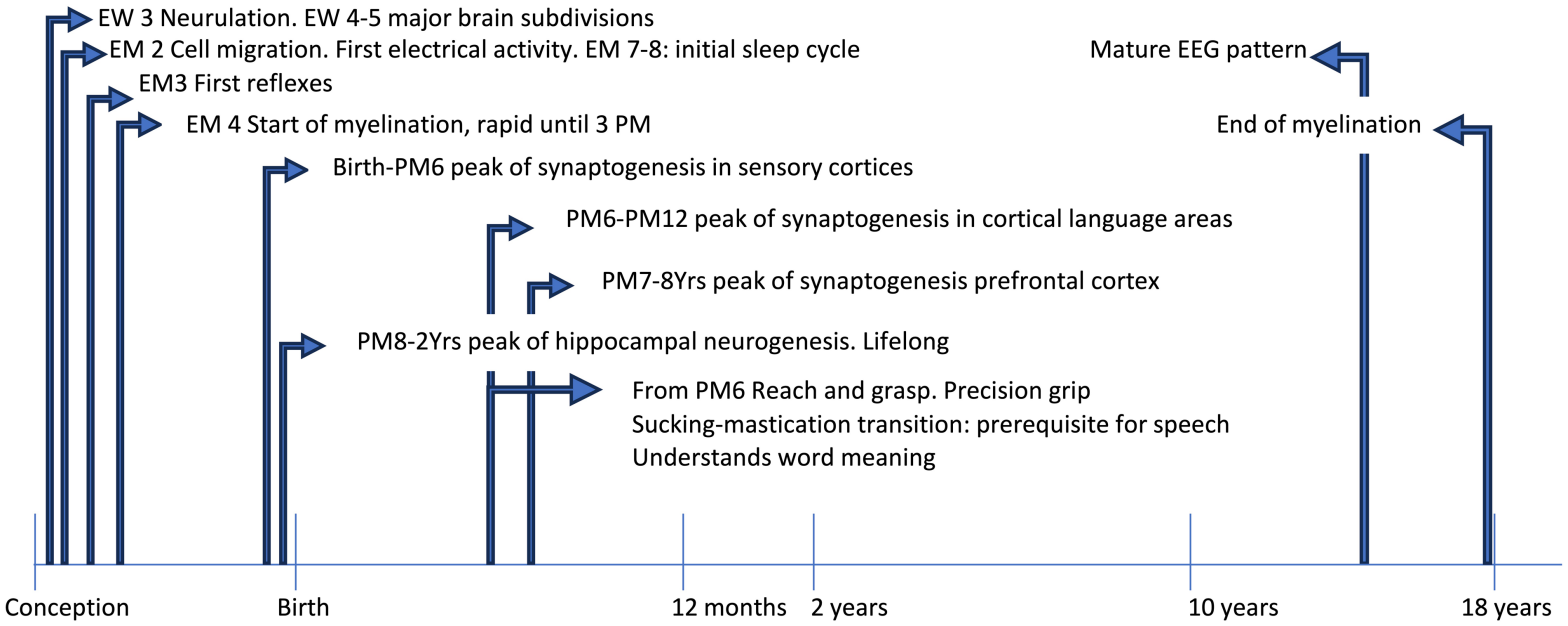
The timeline shows some acquisitions during typical development. EEG, electroencephalographic; EM, embryonic month; EW, embryonic week; PM, postnatal month; Yrs, years.

The study of brain development from pregnancy to adult age has been devoted for years to establishing a fix sequence of events in the morpho-functional development. This vision was the consequence of two concurrent causes, in different fields of knowledge. First, the development of theories of cognitive development in the field of child psychology, during the last century, that described a rather fixed sequence of functional acquisitions from birth onwards, during typical development. This brought forward the underlying idea that both the sequence and timing of acquisition were rather stable and culture-independent, at least in the first phases of development. A revision of the literature on these topics is outside the scope of this review: the readers can refer to the seminal works of leaders in the field, like Jean Piaget and Lev Semënovič Vygotskij. Second, in the emerging field of neuroscience, most data on brain development were from histology, while physiological recordings were limited to certain ages and were mostly not intended to detect lifelong changes. This had the consequence of transmitting the idea that fixed steps of structural and functional development are reached at precise times, with an invariant sequence and limited variability in timing. Some notable exceptions included the studies on plasticity of the visual cortex, that led David Hubel and Torsten Wiesel to share the Nobel prize in 1991. However, technical advances like functional neuroimaging and powerful electrophysiological registration, coupled to the enhanced information processing and miniaturization of devices, led in the last three decades to a paradigm shift in neurosciences. This happened for the inspiring works of Penfield on cortical registrations (Penfield and Boldrey, 1937; Penfield and Jasper, 1954), leading to the definition of brain functional maps (the so-called ‘homunculus’), that now appear more and more plastic even in adults. It is becoming increasingly clear that any function of the brain emerges from a complex network of interactions between gene expression and environmental inputs, at the micro- and macro-scale. Under usual circumstances, moving from one step to the other occurs along a phylogenetically defined best-fit pathway, common to most mammals, that goes from sensory-motor to social and cognitive development, linked to the maturation of specific brain areas and networks. As such, any anomaly may hamper the typical developmental scheme (see Figure 1) and triggers a wealth of downstream effects aimed at fixing the path, with outcomes that may be fitting or not.

However, during the developmental path (summarized in Table 1) some critical periods of particular sensitivity and plasticity may be delimited, which span through infancy (for example: language acquisition) and extend to adolescence, in particular for some functions like memory and social stress management (Fuhrmann et al., 2015). Actually, time is a critical factor in development, since the susceptibility to some influences may dramatically vary, as well as the consequences at different time frames, including biochemical, electrical, genomic and epigenetic mechanisms, up to the effects at the level of development of the organism, which may be lifelong (Boyce et al., 2020).

**Table 1:** Summary of the main developmental steps during infancy.

Age	Stage of development	Main changes	Developmental abilities
Prenatal period	Fetal development	Structural features of the brain Neurons and synapses start to mature from the spinal cord Gyri and sulci formation First synapses and myelination	First movement of the fetus Sensory development of the fetus
First two years	Sensory-motor stage	Gradual development of prefrontal cortex and cerebellum Fusiform gyrus (visual attention) Myelination increase Visual/auditory cortex Increased brain connectivity Experience dependent synapse formation	0–3 months Development of visual and sound perception: turns head toward speakers and follows face Emerging head control More controlled movements (hands in the middle line) Looks at adult face/Respond to facial expression Smile at response
		Continue development of motor cortex Visual/auditory cortex Experience-dependent synapse formation	3–6 months When pulled to sitting, holds head in line with body Reaches side position Mouths toys Reaches and grasps toys Looks toward noises Smiles in response to speakers
		Connectivity between the amygdala and bilateral anterior insula (fear expression); experience dependent synapse formation	6–9 months Sits alone and extends arm if falling to the side Crawls forward on belly Picks up object easily and transfers them from hand to hand Expresses emotional states Responds differently to caregivers and strangers Looks at objects and family members when named Imitates facial expressions, actions and sounds
		Angular Gyrus/Broca area maturation	9–12 months Cruises holding on the furniture Stands alone momentarily Imitates actions (claps hands and waves on command) Turns when called by name Gives objects by request Expresses emotions and affections Articulates most speech sounds
		Angular Gyrus/Broca area refinement (receptive language and speech production)	12–24 months Walks forward and backwards Walks up and down stairs with assistance Demonstrates use of everyday items Language fast development Plays alone and with peers
2–7 years	Pre-operational stage	Synaptic density in the prefrontal cortex reaches its peak Frontal and temporal lobes (executive function and emotional regulation)	Language improvement Increased cognitive abilities Uses symbols in play and pretending Executive functions (attention control, memory self-regulation, emotional regulation)

Here we review the recent literature on the development of the human brain and its susceptibility to both negative and positive environmental influences. A search on PubMed was done on March 24, 2023 with the following terms: BRAIN and PLASTICITY and CHILD and DEVELOPMENT, in any field, with no filters for language or year of publication: 1705 papers were retrieved. By applying the filters: Meta-Analysis, Review and Systematic Review 573 papers were excluded to give 1,132 papers. The goal was to focus on healthy brain development, hence abstracts were read and evaluated to exclude papers exclusively or mainly related to Autism (*n* = 75), other diseases (*n* = 376) or out of focus, including studies done on cells or animals, or retracted studies (*n* = 311). The resulting 370 articles were evaluated, sorting out those describing studies on the morphological and functional normal development of the brain, stress effects on the brain and music training effects on the brain. Articles related to second language learning or other manipulations were excluded, as well as comment articles or introduction to issues, or duplicate publications (same authors, title or content, also in different languages). The 125 resulting relevant articles are reviewed here.

## 2. Morphological and functional development of the brain

### 2.1. Structural changes across development

A large longitudinal study addressed the question of whether cognitive improvement preceded, accompanied or followed the changes in the thickness and surface area of the cortex from infancy to adulthood, when an association between cortical measurements and cognitive performance is apparent: being the rate of change at each measurement predictive of subsequent changes, it was concluded that structural changes in the cortex are related to cognitive performance and *vice-versa*, without a clear sequence in any measure (Estrada et al., 2019). Cortical thickness has been also specifically linked to different neurodevelopmental and psychiatric disorders (Patel et al., 2021).

Normative structural data in the first 6 years of life highlight a generalized thinning of cortex, with the exception of the occipital areas, which first decrease and then become thicker (Remer et al., 2017). Cortical thinning is mostly related to pyramidal neurons, astrocyte and microglia marker genes (Shin et al., 2018). Also, during the first 5 years of life, genes in cortical neurons change dramatically their methylation status, while later changes are much reduced (Price et al., 2019).

Structural maturation of the frontal cortex, crucial for executive functions, requires a prolonged postnatal development, which involves also the migration and integration of newly formed inhibitory interneurons (Paredes et al., 2016). Interestingly, inhibitory control training in children shows larger effects that in adolescent, both on the efficiency of behavioral control and in the structure of some subdivisions of the inferior frontal gyrus in the prefrontal cortex (Delalande et al., 2020). Maturation of cortical circuits are also echoed by biochemical fingerprinting in specific cell types. Neural cell adhesion molecule (NCAM) isoforms are implicated in cell migration, axonal growth and synaptic plasticity, besides schizophrenia. As determined in post-mortem samples, they peak at different times during development in the various areas, from fetal/early infancy to late adolescence, supporting specific roles in neural circuitry formation, emerging at precise developmental timepoints (Cox et al., 2009). Also, connections between areas mature with time: for example, at 9 months the connections over longer distance are emerging, while local network connectivity decreases (Damaraju et al., 2014). Throughout development, structural and functional connectivity develop with non-linear trajectories, which also differ for the various areas, starting *in utero* and extending to late adolescence (Vandewouw et al., 2021). Increased connectivity boosts the emergence of executive and cognitive functions, with a differential contribution of the striatum, which improves cognitive functions in childhood through increase in the cortico-cortical connections, while its effects on the executive functions appear less age-related (Darki et al., 2020). During adolescence, myelin microstructure refines to increase the speed of electrical signal transmission, at the expenses of diminished plasticity, and mature first in sensorimotor areas and then in associative areas (Baum et al., 2022).

### 2.2. Development of sensory-motor functions

In the striate cortex, functional plasticity mirrors the number of synapses that peaks in the first year of age and is rapidly refined and reduced in the subsequent pre-school years (Huttenlocher and de Courten, 1987). Early imaging studies reported a decrease in grey matter of frontal and parietal cortices during adolescence (Jernigan et al., 1991), while enzymes related to cholinergic and glutamatergic neurotransmission vary across the entire lifespan, in specific areas (Court et al., 1993). Notably GABA-ergic neurotransmission show a protracted period of postnatal refinement, spanning the first years of life and possibly accounting for the protracted plasticity of visual areas, which allows therapeutic interventions (Murphy et al., 2005).

Also, in the primary motor cortex GABAergic interneurons mature after childhood, fostering plasticity and motor learning (Walther et al., 2009). Contrary to findings in monkeys, in humans the corticospinal projections start connecting with spinal cord at 24 post-conception weeks, between 2 and 4 postnatal months the spontaneous activity becomes more coordinated between limbs, yet functional control of distal effectors is reached much later, between 6 and 12 months of age, to support goal-directed movements (Eyre et al., 2000; Kanemaru et al., 2012).

Movement of the hand requires the identification of targets, which is usually based on vision: already 2 days after birth, newborns may be trained to discriminate kinematic patterns of biological movement, characterized by subsequent acceleration/deceleration, even if they spontaneously do not (Craighero et al., 2020). Already at 5–6 months, the movement of the hand is typically directed to a person or to an object: this specificity is missing in children not sharing typical developmental paths (Ouss et al., 2018). Sensorimotor coordination accuracy improves in primary school children while sensorimotor integration relies on subcortical circuits maturing at a later stage, towards adulthood (Savion-Lemieux et al., 2009). On the other hand, in children and adults, observation of an action activates the same mirror-neurons system (premotor cortex-inferior frontal gyrus and posterior parietal lobe), but with a more widespread and more bilateral activation in children than in adults (Biagi et al., 2016). Human movement develops through the progressive control of tools use, which requires a complex dynamic between body size representation and sensory inputs, so that only in late puberty the body representation acquires adult features and may rely on proprioception instead of visual perception (Martel et al., 2021). Of note, children do not adapt as adults to tactile stimuli, until 8–10 years of age, suggesting a different sensory experience in addition to a different stimulus processing (Domenici et al., 2022).

Expert visual processing of face is lateralized to the right hemisphere and requires visual input during infancy to become fully operational, suggesting protracted need for stimulation (Le Grand et al., 2003), yet face specialization starts before reading acquisition an impinges on the decreased cortical responses to the other stimuli (Cantlon et al., 2011), as well as on higher glutamate relative to GABA levels in the inferior frontal gyrus (Cohen Kadosh et al., 2015). Similarly, movement-directed visual attentional shifts for actions appears already at 7 months, suggesting concomitant maturation of visual and attentional systems (Daum et al., 2016). Later on, during school-age period, the increase in activity of the adrenal gland with dehydroepiandrosterone (DHEA) surge, promotes the concomitant maturation of amygdala with occipital lobe, related to visual awareness, parietal lobe, related to visuomotor abilities, and frontal lobe, related to attention (Nguyen et al., 2016). Apparently, DHEA in childhood helps optimization of attentional and working memory functions but may impair the processing of spatial cues by reducing the connections from hippocampus to cortex (Nguyen et al., 2017).

Functional lateralization requires the maturation of corpus callosum, which has a critical refinement period after 6 years of age, thus affecting language transfer between the two hemispheres (Westerhausen et al., 2011). Myelinization is indeed critical for full functional maturation, but while myelin turnover is fast, the number of oligodendrocytes in the corpus callosum is stable from childhood, with a yearly exchange rate of only 1 out of 300 (Yeung et al., 2014). Prolonged maturation and myelin plasticity appear related also to increased functional cognitive ability already by 3 years of age (Deoni et al., 2016). In the first 2 years of life myelination and microstructural properties of glia appear related to cognitive abilities, with protracted development associated to better performance in cognitive and language tasks (Girault et al., 2019). Also, cortical thickness in the first 2 years appears related to cognitive abilities, yet the contribution of gestational age and maternal education may overcome structural differences (Girault et al., 2020).

More complex sensory functions, including multisensory integration, appear subsequently after middle childhood (Ernst, 2008), and their fine-tuning appears complete by 14 years (Brandwein et al., 2011). Cognitive mathematical abilities may be related to white matter in the left parietal lobe (Matejko et al., 2013) and better outcomes of intense math training rely on left perisylvian tracts plasticity (Jolles et al., 2016a), with a specific effect on connectivity of the intraparietal sulcus, but not of the angular gyrus, with hippocampus, lateral prefrontal and ventral temporo-occipital cortex (Jolles et al., 2016b). The intraparietal sulcus content in glutamate and GABA appears developmentally linked to math abilities (Zacharopoulos et al., 2021). Similarly, abacus training in primary school children improves performance and executive functions by modulating frontoparietal activation (Wang et al., 2017), and appears linked to the volume of fusiform gray matter (Zhou et al., 2022). In the first 2 years of primary school, better outcomes in mathematical abilities are associated with specific changes in some cortical areas, in detail: arithmetic abilities are linked to folding change in the right intraparietal sulcus, and thickness changes in right temporal lobe and left middle occipital gyrus, while visuospatial abilities are linked to right superior parietal thickness, and other frontal areas in the right hemisphere (Kuhl et al., 2020). However, complex visuospatial tasks elicit strong bilateral parietal activation in both adults and children from age 5 onwards (Ferrara et al., 2021).

Quantitative differences in cognitive abilities, operationally defined as a higher IQ, appear linked to a prolonged sensitivity to environmental influences (Brant et al., 2013) while its relationships to cortical thinning and surface area in late childhood appears controversial (Burgaleta et al., 2014; Schnack et al., 2015). Functional control for cognitive functions emerges at a later time, for example executive attention is linked to planning and inhibitory control, and allows the development of self-regulation (Rueda et al., 2005). Functional plasticity appears high in late childhood also for memory function (Brehmer et al., 2007). Emotional regulation steers impulsivity in the context of prospective thinking: it appears in late childhood and is related to insula thickness (Churchwell and Yurgelun-Todd, 2013). As outlined above, amygdala development is crucial in managing the emotional reactivity: it is noteworthy that the paralaminar nuclei of amygdala host a population of immature cells that slowly develops through childhood and adolescence into excitatory neurons but still persists even in old age, suggesting a protracted plasticity in this area (Sorrells et al., 2019).

The developmental trajectory goes through a reduction in modularity and local efficiency of brain processing, while increasing global efficiency. This increase in the efficiency of global processing, linked to functional maturation, initially affects sensorimotor areas. At variance, associative and paralimbic areas show a protracted plasticity during late childhood, which may account for peripubertal behavioral modulation (Khundrakpam et al., 2013). In school-age children, enhanced brain modularity may also prepare for disclosing effects of physical activity on cognitive and executive functions (Chaddock-Heyman et al., 2020).

### 2.3. Sleep to grow

Already during the first year of life, the pattern of night sleep and awakening may predict typical and atypical cognitive trajectories (Pisch et al., 2019), while slow waves propagation during the night, which depends on brain connectivity, is reduced in toddlers compared to older children (Schoch et al., 2018). Also, the decline in slow-wave non-REM sleep activity is steeper during adolescence, in caudal-rostral direction, suggesting late functional reorganization following structural synaptic pruning (Feinberg et al., 2011). At variance, local increase in slow-wave activity over the right parietal areas, related to visuomotor-dependent plasticity, is higher in children (Wilhelm et al., 2014), as it is the slow-wave increase in left frontoparietal areas after working memory training (Pugin et al., 2015). Around 1 year of age, sleep spindles appear to be related to semantic generalization of words (Friedrich et al., 2015), while in school-age children the learning-dependent hippocampal activity and sleep-related frontal activity do change at a faster rate than in adults (Urbain et al., 2016). Children also show the largest overnight slope change in slow waves, which may be related to the increased plasticity of children brain (Jaramillo et al., 2020). Slow waves are generated by corticocortical connections while spindles results from thalamocortical activity and in adolescents appear modulated by genetic background in posterior areas, while spindles in anterior areas are more sensitive to environmental factors (Rusterholz et al., 2018). Interestingly, the larger modulation of slow-wave activity during night in children is not accompanied by the change in glutamate/glutamine which is apparent in adults, pointing to different biochemical pathways in children (Volk et al., 2019). Another feature of human EEG activity is the alpha oscillation, which shows a maturation during childhood, most apparent for the aperiodic component: this is related to increased thalamocortical connections and attentional performance (Tröndle et al., 2022).

## 3. How environment may interact with developmental trajectories

The effect of environment on development can be positive or negative from the very beginning of pregnancy throughout postnatal life. Among negative environmental regulations with a heavy societal impact, the effect of pre-natal alcohol exposure has long been studied in both animals and humans. Several studies have documented the long-lasting effects of maternal alcohol consumption on both the structure and functions of the developing brain and ultimately child fitness. Despite the detailed discussion of this topic is outside the scope of this Review, we highlight that early longitudinal studies proved the adverse effects of heavy drinking during pregnancy on the morpho-functional development, in particular in the parietal cortex (Lebel et al., 2012) and in the development of white matter in relation to executive functions (Gautam et al., 2014).

Also, other environmental stimuli or their absence may interfere with the development of structural features and functional acquisitions during postnatal development. In order to develop harmonic abilities, the interaction with environment may foster or hinder functions after their appearance. As an example, children with blind parents normally show the eye contact from birth, which usually is a common means of communication, but by age 6 months onwards they display progressively less attention to gaze processing, even if this is not related to impairments in social or cognitive abilities, suggesting that even ‘obvious’ abilities require a strong social/environmental input and practice to be fully operational (Senju et al., 2015). Hence, the neuroscientific literature provides several lines of evidence that support the steering role of environmental stimuli in early development, with durable effects in different areas, both anatomical and functional, including cognitive, emotional, and social abilities.

### 3.1. Case studies

Here we briefly introduce two examples, one of negative (stress) and one of positive (music training) environmental influences on the development of the brain and its functions, to highlight how much we as adults are responsible for the life paths of future generations. In order to include articles from different countries, socioeconomical status and cultures, we focused on two modulators that were less likely linked to these factors. Stressful environmental conditions like low socioeconomical status, trauma or neglect share similar characteristics among different cultures. Also, music and music training are widespread in all cultures, and include learning sensory-motor abilities which makes them less dependent on overall cognitive abilities or higher socioeconomical status than, for example second language training. Thus, music training appears a robust example to explore in this review.

### 3.2. The role of stress

Stress may profoundly impact on neurodevelopmental trajectories, at different ages, by means of the different neural, endocrine, neuroendocrine, metabolic and immune responses. Notably, the brain itself is the target of stress hormones, that shape the brain stress response, tuned to plasticity, a double-edged sword that may either blunt or enhance adaptive responses, modifying the vulnerability to early life stressors. Under this respect, the concept of resilience includes the processes leading to positive adaptation to relevant traumas or adverse challenges. Early rehabilitation and care, including proprioceptive stimulation as sensory-tonic stimulation and kangaroo care, coupled to parenting support may influence the development of very preterm infants (Guittard et al., 2023), while severe maltreatment or abuse impairs functional and structural brain development, thus representing a relevant threat for the single person and a significant cost for society. Hence, boosting resilient responses in high-risk persons may promote neural and neuroendocrine plasticity to decrease maladaptive or even psychopathological responses (Cicchetti, 2010). In the first 2 years of life, elicited imitation task as a tool to investigate declarative memory, reveals that neglected children do not receive maternal feedback while abused children do, leading to a loss of plasticity in neglected children, while increased imitation in abused infants possibly leads them to increased cognitive but decreased social competence (Cheatham et al., 2010). The brain-derived neurotrophic factor (BDNF) is involved in synaptic plasticity and is differentially expressed in childhood (Sterner et al., 2012). Interestingly, the exposure to unfavorable environment leads to depression in persons carrying the Val66Met BDNF polymorphism (Comasco et al., 2013), with Val carriers of the same polymorphism more prone to self-injurious behavior (Bresin et al., 2013), while childhood abuse in Met carriers results in poorer cognitive performance and brain anomalies, including larger lateral ventricles and reduced right hippocampus (Aas et al., 2013). The same polymorphism appears to impact stress experience more at late stages (Lehto et al., 2016). BDNF methylation in adolescent brain is related to neighborhood disadvantage and thinner lateral orbitofrontal cortex (Wrigglesworth et al., 2019). Children outcome on different measures was linked to parenting quality, but also to BDNF status and genes involved in dopamine and serotonin neurotransmission, that appear to convey some vulnerability to environmental stress, fostering the vision of a continuum of general traits to describe the responses to stress, instead of two susceptibility traits (e.g., the orchid/dandelion duality) leading to different responses (Zhang et al., 2021). By widening the analysis to the BDNF gene network, it appeared that this network interacted with adverse prenatal conditions to affect later cognitive development, so that a high BDNF network score coupled to high prenatal adversity resulted in slower cognitive development and grey matter density in associative cortical areas (de Mendonça Filho et al., 2021).

Material hardship linked to poverty may lead to different amygdala-prefrontal cortex connectivity in late infancy, and leads to reduced amygdala-orbitofrontal cortex connections in adolescents, also related to anxiety and depression, indicating some preferential windows of plasticity for targeted supporting interventions (Hardi et al., 2022).

Low socioeconomic resources may impair visual working memory, as a proxy for cognitive abilities, and related brain activity in the left frontal cortex of children up to 4 years old (Wijeakumar et al., 2019). The socioeconomical status may result in differential exposure to language or stress, which may be the bases for the differences in hippocampus and amygdala seen in socioeconomically disadvantaged children, with an additional contribution of age, inducing additional differences in the left superior temporal and inferior frontal gyri (Noble et al., 2012). Similar reductions in amygdala and hippocampus were detected in children experiencing early life stress in the form of physical abuse, early neglect or low socioeconomic status (Hanson et al., 2015). Child abuse appears also to affect the morphological complexity of the prefrontal cortex and to increase recruitment of perineuronal networks, mediated by oligodendrocyte precursors, that lead to decreased plasticity (Tanti et al., 2022).

A large study confirmed that trauma exposure resulted in adolescent thinner superior frontal gyri and right amygdala and larger cingulate cortices (Jeong et al., 2021). Child maltreatment results in increased Cornu Ammonis (CA) 4 subfield of hippocampus, most apparent in males, while larger CA1 is associated with late-onset psychopathology, suggesting that maltreatment differentially affects hippocampal subfields, which may precede the appearance of psychopathology (Whittle et al., 2016). The level of self-perceived stress is also associated with smaller hippocampal volume in adolescents (Piccolo et al., 2018), and in a longitudinal study, attachment dimensions like anxiety and avoidance were linked to larger decreases in prefrontal and anterior temporal cortices in adolescent brain (Puhlmann et al., 2023).

In adolescents, post-traumatic stress disorder, as a result of altered fear regulation, is linked to decreased grey matter volume in the centromedial and basolateral amygdala, whose connectivity with left orbitofrontal and subcallosal cortices is increased, while connections to the right cingulate and prefrontal cortices appear less strong (Aghajani et al., 2016).

Volumetric correlations among different areas indicate that prenatal stress but not childhood trauma may de-couple amygdala growth from the development of other regions involved in emotional processing (Mareckova et al., 2022). Early childhood deprivation induces long-term modifications, apparent in adult white matter tracts, in particular of the limbic circuits and long-ranging association fibers, while the microstructural organization appears not altered (Mackes et al., 2022). Also, epigenetic changes in some genes associated to child abuse may enhance the risk of child depression (Weder et al., 2014), and regions of low methylation differentiated children receiving less tactile contact (Moore et al., 2017). Lastly, genome-wide mapping fished out some loci linked to postnatal stress and subcortical structures like caudate and accumbens nuclei, which have a role in neuronal plasticity and neurodevelopmental disorders, however causality remains to be ascertained (Bolhuis et al., 2022).

### 3.3. Music training – positively steering development

Highlights for positive experience-related brain plasticity stem from the effects of music training in infancy, because of the worldwide diffusion of music across all human cultures, paralleled by a widespread training at young age, which is however not diffuse to all persons. Because of the multimodal nature and prolonged practice of instrument or voice, music training appears well suited to exploit plastic capabilities of the brain.

Adult musicians show increased sound discrimination, as a result of training. Early music training enhances children performance in specific musical skills like melody discrimination (Ireland et al., 2019). After 2 years of training in school age children, an improvement in tonal discrimination and increased maturity of auditory processing are apparent (Habibi et al., 2016). In 9 to 15 years old trained or untrained subjects, cognitive flexibility was linked to sound discrimination performance, which was more apparent in music-trained group (Saarikivi et al., 2016).

Instrument practice allows a regional-specific increase in the organization of the pyramidal tract already in childhood (Bengtsson et al., 2005) and extend to cognitive abilities underlying musical training, initially on more closely related fields (Schlaug et al., 2005). Long lasting effects on motor performance appear stronger if the music practice starts before age seven, even if the amount of training was similar, pointing to the existence of a sensitive period in infancy (Penhune et al., 2005).

Interestingly, even relatively a short period (9 months) of music training may produce benefits for pitch processing in music but also in language, showing that cognitive benefits extend over different cognitive domains, by modifying their neural substrates and related pattern of brain activity (Moreno et al., 2009). Musical and linguistic syntactic abilities may be learned through similar processes in early infancy and about age 4–5, music training affects timbre identification and improve language abilities, like morphologic rule formation and memory for words, showing that training effects extend beyond the music domain (Marin, 2009). Notably, increased right brain activity due to human voice processing is related to intelligence in toddlers and school-age children (An et al., 2020).

Preschool children benefit even from short (20 days) music training, whose effects spill over to verbal intelligence and executive function tasks (Moreno et al., 2011). Over 2 years of training around 8 years old, speech segmentation skills improve more than in untrained children suggesting therapeutic strategies for children with language impairments and related learning difficulties (François et al., 2013).

In preschool children, music or second language training induce long-lasting improvement in processing of the trained sounds and increase suppression of untrained, non-relevant sounds, as shown by event-related potentials (Moreno et al., 2015).

By exploring the brain structure, aptitude to music in school-age children is related to pre-training structural organization of the right corticospinal tract while the corpus callosum structure appears more linked to tonal ability (Zuk et al., 2022).

Increased pitch discrimination is related to larger auditory regions in both untrained and music-trained adults and children, while in musicians it is also associated to larger inferior frontal gyrus (Palomar-García et al., 2020).

After only 15 months of practice, music training may induce structural changes in the brain, directly related to improvements in auditory and motor skills (Hyde et al., 2009). Apparently, starting musical training before age 7 changes white matter connectivity, more robustly in the isthmus of corpus callosum: therein, fractional anisotropy, related to myelinization, is linked to both age of starting the training and sensorimotor synchronization performance (Steele et al., 2013). On the other hand, the benefits of music appear to extend also to prenatal age, since in preterm infants exposed to musicotherapy, the maturation of white matter improved in acoustic radiations, claustrum and uncinate fasciculum, and also amygdala volumes increased, suggesting improved acoustic and emotional processing, compared to non-exposed preterm and full-term babies (Sa de Almeida et al., 2020). The age of onset is critical for structural changes to appear: focusing on exposure to a second language and music, it emerged that the arcuate fasciculus, which participates in both music and language activities by linking areas of the dorsal auditory pathway, is sensitive to second language in the left hemisphere, while in the right one it changes according to music exposure (Vaquero et al., 2020). Hence, the structure of different areas is selectively modified according to the type of experience mostly in early infancy.

## 4. Development is socially modulated

A relevant, yet underappreciated, issue in developmental neuroscience is the social nature of our species. In a study involving both parents and children, focused on the intergenerational transmission of sociality, the parents’ limbic, embodied simulation and mentalizing networks appeared linked to the use of strategies for children’s emotion regulation, suggesting a strong link between parent–child interactions and later child social life (Abraham et al., 2016). However maternal influence starts prenatally, since maternal stress (e.g., pandemic-related) affects 3 months infants’ regulatory capacity (Provenzi et al., 2021), and also extends to calibration of growth rate and timing of sexual development, by affecting postnatal testosterone levels in infants (Corpuz, 2021). Maternal depression during pregnancy appears to influence the development of amygdala, by interacting with the canonical transforming growth factor-beta (TGF-β) signaling pathway (Qiu et al., 2021).

In school-age children, parental praise as a positive parenting style may result in increased openness to experiences and carefulness, together with increase in gray matter in the posterior insula, which is involved in empathy modulation due to the connections with amygdala (Matsudaira et al., 2016). By using fMRI-based neurofeedback, it was possible to demonstrate that both children and adolescents can learn to upregulate amygdala function, suggesting a possible tool to act on regulation of emotional reactivity (Cohen Kadosh et al., 2016).

## 5. Conclusion

The possibility of non-invasive exploration of the living brain also in children, emerged in the last years, led to a dramatic increase in our understanding of development of the brain and its functions, in the context of the growing body. The development of cognitive, emotional, and social behaviors appears more along a continuum than limited to narrow developmental windows, while certain attainments may be specific to some developmental periods (Guyer et al., 2018). Out of the laboratory, the increasing knowledge of the developmental processes led to a paradigm shift in the approach to children, in the context of parental relationships and pedagogical approaches, up to inform the political processes. Actually, sharing knowledge accumulating through dedicated studies on brain development and the increasing evidence about the long-lasting functional outcome of environmental modifications, may serve to raise consciousness about the actions to undertake to provide support and care to fragile children. While awareness of environmental risks for development and overall health is increasing (Chesney and Duderstadt, 2022), a widespread knowledge of the risks and possibilities for external actions to drive children development is still on the way. The possibility of early detection of child needs even with primary pediatric care and support to the family increases the chances of steering cognitive, emotional and social development towards positive outcomes (Williams and Lerner, 2019). Addressing adverse childhood experiences requires fostering health and educational services to promote the foundation of lifelong health, with the necessary inclusion of family (Bethell et al., 2017). Assistance for families, starting from maternal health, and for communities will provide supporting relationships to lay the foundation of resilience throughout life (Traub and Boynton-Jarrett, 2017). This should be declined across different cultures and is particularly relevant for children with additional requirements, like neurodevelopmental disorders (Bannink Mbazzi and Kawesa, 2022). Including constructs like ‘neuroplasticity’ in the educational trajectories led to an empowerment of the main actors, children, parents and teachers by fostering executive functions (Choudhury and Wannyn, 2022). The contribution of widespread schooling on the social construction of cognition and neurocognitive development has long been appreciated (Baker et al., 2012). In these last years, programs have been designed to support selective attention in children from low socioeconomic status with some genotypes which may represent a risk factor (Isbell et al., 2017). However, cognitive development is only one side of the coin: the role of social regulation of development, starting from parents to the group of peers needs to be recognized and actively included in political long-sighted plans. More can be done on the bases of the recent data on the involvement of social processes in the development of self-regulatory processes, to improve both personal development and society.

## Author contributions

CM-C: Conceptualization, Data curation, Funding acquisition, Investigation, Writing – original draft. GS: Writing – review & editing.

## References

[ref1] Aas M. Haukvik U. K. Djurovic S. Bergmann Ø. Athanasiu L. Tesli M. S.. (2013). BDNF val66met modulates the association between childhood trauma, cognitive and brain abnormalities in psychoses. Prog. Neuro-Psychopharmacol. Biol. Psychiatry 46, 181–188. doi: 10.1016/j.pnpbp.2013.07.008, PMID: 23876786

[ref2] Abraham E. Hendler T. Zagoory-Sharon O. Feldman R. (2016). Network integrity of the parental brain in infancy supports the development of children’s social competencies. Soc. Cogn. Affect. Neurosci. 11, 1707–1718. doi: 10.1093/scan/nsw090, PMID: 27369068 PMC5091682

[ref3] Aghajani M. Veer I. M. van Hoof M. J. Rombouts S. A. van der Wee N. J. Vermeiren R. R. (2016). Abnormal functional architecture of amygdala-centered networks in adolescent posttraumatic stress disorder. Hum. Brain Mapp. 37, 1120–1135. doi: 10.1002/hbm.23093, PMID: 26859310 PMC6867491

[ref4] An K. M. Hasegawa C. Hirosawa T. Tanaka S. Saito D. N. Kumazaki H.. (2020). Brain responses to human-voice processing predict child development and intelligence. Hum. Brain Mapp. 41, 2292–2301. doi: 10.1002/hbm.24946, PMID: 32090414 PMC7267979

[ref5] Baker D. P. Salinas D. Eslinger P. J. (2012). An envisioned bridge: schooling as a neurocognitive developmental institution. Dev. Cogn. Neurosci. 2, S6–S17. doi: 10.1016/j.dcn.2011.12.001, PMID: 22682912 PMC4507825

[ref6] Bannink Mbazzi F. Kawesa E. S. (2022). Impairments of the brain: global south perspectives on childhood neurodevelopmental disability. Dev. Med. Child Neurol. 64, 1193–1201. doi: 10.1111/dmcn.15253, PMID: 35524350 PMC9545297

[ref7] Baum G. L. Flournoy J. C. Glasser M. F. Harms M. P. Mair P. Sanders A. F. P.. (2022). Graded variation in T1w/T2w ratio during adolescence: measurement, caveats, and implications for development of cortical myelin. J. Neurosci. 42, 5681–5694. doi: 10.1523/JNEUROSCI.2380-21.2022, PMID: 35705486 PMC9302463

[ref8] Bengtsson S. L. Nagy Z. Skare S. Forsman L. Forssberg H. Ullén F. (2005). Extensive piano practicing has regionally specific effects on white matter development. Nat. Neurosci. 8, 1148–1150. doi: 10.1038/nn1516 16116456

[ref9] Bethell C. D. Solloway M. R. Guinosso S. Hassink S. Srivastav A. Ford D.. (2017). Prioritizing possibilities for child and family health: an agenda to address adverse childhood experiences and Foster the social and emotional roots of well-being in pediatrics. Acad. Pediatr. 17, S36–S50. doi: 10.1016/j.acap.2017.06.002 28865659

[ref10] Biagi L. Cioni G. Fogassi L. Guzzetta A. Sgandurra G. Tosetti M. (2016). Action observation network in childhood: a comparative fMRI study with adults. Dev. Sci. 19, 1075–1086. doi: 10.1111/desc.12353 26537750

[ref11] Bolhuis K. Mulder R. H. de Mol C. L. Defina S. Warrier V. White T.. (2022). Mapping gene by early life stress interactions on child subcortical brain structures: a genome-wide prospective study. JCPP Adv. 2:12113. doi: 10.1002/jcv2.12113, PMID: 36777645 PMC7614163

[ref12] Boyce W. T. Sokolowski M. B. Robinson G. E. (2020). Genes and environments, development and time. Proc. Natl. Acad. Sci. U. S. A. 117, 23235–23241. doi: 10.1073/pnas.2016710117, PMID: 32967067 PMC7519332

[ref13] Brandwein A. B. Foxe J. J. Russo N. N. Altschuler T. S. Gomes H. Molholm S. (2011). The development of audiovisual multisensory integration across childhood and early adolescence: a high-density electrical mapping study. Cereb. Cortex 21, 1042–1055. doi: 10.1093/cercor/bhq170 20847153 PMC3077428

[ref14] Brant A. M. Munakata Y. Boomsma D. I. Defries J. C. Haworth C. M. Keller M. C.. (2013). The nature and nurture of high IQ: an extended sensitive period for intellectual development. Psychol. Sci. 24, 1487–1495. doi: 10.1177/0956797612473119, PMID: 23818653 PMC4511162

[ref15] Brehmer Y. Li S. C. Müller V. von Oertzen T. Lindenberger U. (2007). Memory plasticity across the life span: uncovering children’s latent potential. Dev. Psychol. 43, 465–478. doi: 10.1037/0012-1649.43.2.465, PMID: 17352553

[ref16] Bresin K. Sima Finy M. Verona E. (2013). Childhood emotional environment and self-injurious behaviors: the moderating role of the BDNF Val66Met polymorphism. J. Affect. Disord. 150, 594–600. doi: 10.1016/j.jad.2013.01.050, PMID: 23541842

[ref17] Burgaleta M. Johnson W. Waber D. P. Colom R. Karama S. (2014). Cognitive ability changes and dynamics of cortical thickness development in healthy children and adolescents. NeuroImage 84, 810–819. doi: 10.1016/j.neuroimage.2013.09.038, PMID: 24071525 PMC3888797

[ref18] Cantlon J. F. Pinel P. Dehaene S. Pelphrey K. A. (2011). Cortical representations of symbols, objects, and faces are pruned back during early childhood. Cereb. Cortex 21, 191–199. doi: 10.1093/cercor/bhq078 20457691 PMC3000569

[ref19] Chaddock-Heyman L. Weng T. B. Kienzler C. Weisshappel R. Drollette E. S. Raine L. B.. (2020). Brain network modularity predicts improvements in cognitive and scholastic performance in children involved in a physical activity intervention. Front. Hum. Neurosci. 14:346. doi: 10.3389/fnhum.2020.00346, PMID: 33100988 PMC7497763

[ref20] Cheatham C. L. Larkina M. Bauer P. J. Toth S. L. Cicchetti D. (2010). Declarative memory in abused and neglected infants. Adv. Child Dev. Behav. 38, 161–182. doi: 10.1016/b978-0-12-374471-5.00007-6 21207809

[ref21] Chesney M. L. Duderstadt K. (2022). Children’s rights, environmental justice, and environmental health policy in the United States. J. Pediatr. Health Care 36, 3–11. doi: 10.1016/j.pedhc.2021.08.006 34922676

[ref22] Choudhury S. Wannyn W. (2022). Politics of plasticity: implications of the new science of the teen brain for education. Cult. Med. Psychiatry 46, 31–58. doi: 10.1007/s11013-021-09731-8, PMID: 34216345

[ref23] Churchwell J. C. Yurgelun-Todd D. A. (2013). Age-related changes in insula cortical thickness and impulsivity: significance for emotional development and decision-making. Dev. Cogn. Neurosci. 6, 80–86. doi: 10.1016/j.dcn.2013.07.001, PMID: 23921157 PMC6987805

[ref24] Cicchetti D. (2010). Resilience under conditions of extreme stress: a multilevel perspective. World Psychiatry 9, 145–154. doi: 10.1002/j.2051-5545.2010.tb00297.x, PMID: 20975856 PMC2948722

[ref25] Cohen Kadosh K. Krause B. King A. J. Near J. Cohen K. R. (2015). Linking GABA and glutamate levels to cognitive skill acquisition during development. Hum. Brain Mapp. 36, 4334–4345. doi: 10.1002/hbm.22921, PMID: 26350618 PMC4832309

[ref26] Cohen Kadosh K. Luo Q. de Burca C. Sokunbi M. O. Feng J. Linden D. E. J.. (2016). Using real-time fMRI to influence effective connectivity in the developing emotion regulation network. NeuroImage 125, 616–626. doi: 10.1016/j.neuroimage.2015.09.070, PMID: 26475487 PMC4692450

[ref27] Comasco E. Åslund C. Oreland L. Nilsson K. W. (2013). Three-way interaction effect of 5-HTTLPR, BDNF Val66Met, and childhood adversity on depression: a replication study. Eur. Neuropsychopharmacol. 23, 1300–1306. doi: 10.1016/j.euroneuro.2013.01.010 23481907

[ref28] Corpuz R. (2021). The role of maternal environment on calibrating mini puberty in early infant development. Dev. Psychobiol. 63, 800–807. doi: 10.1002/dev.22033, PMID: 32896902

[ref29] Court J. A. Perry E. K. Johnson M. Piggott M. A. Kerwin J. A. Perry R. H.. (1993). Regional patterns of cholinergic and glutamate activity in the developing and aging human brain. Brain Res. Dev. Brain Res. 74, 73–82. doi: 10.1016/0165-3806(93)90085-o, PMID: 8104741

[ref30] Cox E. T. Brennaman L. H. Gable K. L. Hamer R. M. Glantz L. A. Lamantia A. S.. (2009). Developmental regulation of neural cell adhesion molecule in human prefrontal cortex. Neuroscience 162, 96–105. doi: 10.1016/j.neuroscience.2009.04.037 19393299 PMC2739580

[ref31] Craighero L. Ghirardi V. Lunghi M. Panin F. Simion F. (2020). Two-day-old newborns learn to discriminate accelerated-decelerated biological kinematics from constant velocity motion. Cognition 195:104126. doi: 10.1016/j.cognition.2019.104126, PMID: 31731117

[ref32] Damaraju E. Caprihan A. Lowe J. R. Allen E. A. Calhoun V. D. Phillips J. P. (2014). Functional connectivity in the developing brain: a longitudinal study from 4 to 9months of age. NeuroImage 84, 169–180. doi: 10.1016/j.neuroimage.2013.08.038, PMID: 23994454 PMC3881974

[ref33] Darki F. Sauce B. Klingberg T. (2020). Pediatric imaging, neurocognition, and genetics study inter-individual differences in striatal connectivity is related to executive function through fronto-parietal connectivity. Cereb. Cortex 30, 672–681. doi: 10.1093/cercor/bhz117, PMID: 31504278

[ref34] Daum M. M. Wronski C. Harms A. Gredebäck G. (2016). Action perception in infancy: the plasticity of 7-month-olds’ attention to grasping actions. Exp. Brain Res. 234, 2465–2478. doi: 10.1007/s00221-016-4651-3, PMID: 27093869

[ref35] de Mendonça Filho E. J. Barth B. Bandeira D. R. de Lima R. M. S. Arcego D. M. Dalmaz C.. (2021). Cognitive development and brain gray matter susceptibility to prenatal adversities: moderation by the prefrontal cortex brain-derived neurotrophic factor gene co-expression network. Front. Neurosci. 15:744743. doi: 10.3389/fnins.2021.744743, PMID: 34899157 PMC8652300

[ref36] Delalande L. Moyon M. Tissier C. Dorriere V. Guillois B. Mevell K.. (2020). Complex and subtle structural changes in prefrontal cortex induced by inhibitory control training from childhood to adolescence. Dev. Sci. 23:e12898. doi: 10.1111/desc.12898, PMID: 31469938

[ref37] Deoni S. C. O’Muircheartaigh J. Elison J. T. Walker L. Doernberg E. Waskiewicz N.. (2016). White matter maturation profiles through early childhood predict general cognitive ability. Brain Struct. Funct. 221, 1189–1203. doi: 10.1007/s00429-014-0947-x, PMID: 25432771 PMC4771819

[ref38] Domenici N. Tonelli A. Gori M. (2022). The development of adaptation aftereffects in the vibrotactile domain. J. Exp. Psychol. Gen. 151, 3134–3143. doi: 10.1037/xge0001252, PMID: 35696173

[ref39] Ernst M. O. (2008). Multisensory integration: a late bloomer. Curr. Biol. 18, R519–R521. doi: 10.1016/j.cub.2008.05.002 18579094

[ref40] Estrada E. Ferrer E. Román F. J. Karama S. Colom R. (2019). Time-lagged associations between cognitive and cortical development from childhood to early adulthood. Dev. Psychol. 55, 1338–1352. doi: 10.1037/dev0000716, PMID: 30829509 PMC6533129

[ref41] Eyre J. A. Miller S. Clowry G. J. Conway E. A. Watts C. (2000). Functional corticospinal projections are established prenatally in the human foetus permitting involvement in the development of spinal motor centres. Brain 123, 51–64. doi: 10.1093/brain/123.1.51, PMID: 10611120

[ref42] Feinberg I. de Bie E. Davis N. M. Campbell I. G. (2011). Topographic differences in the adolescent maturation of the slow wave EEG during NREM sleep. Sleep 34, 325–333. doi: 10.1093/sleep/34.3.325, PMID: 21358849 PMC3041708

[ref43] Ferrara K. Seydell-Greenwald A. Chambers C. E. Newport E. L. Landau B. (2021). Development of bilateral parietal activation for complex visual-spatial function: evidence from a visual-spatial construction task. Dev. Sci. 24:e13067. doi: 10.1111/desc.13067, PMID: 33226713 PMC8594159

[ref44] François C. Chobert J. Besson M. Schön D. (2013). Music training for the development of speech segmentation. Cereb. Cortex 23, 2038–2043. doi: 10.1093/cercor/bhs180 22784606

[ref45] Friedrich M. Wilhelm I. Born J. Friederici A. D. (2015). Generalization of word meanings during infant sleep. Nat. Commun. 6:6004. doi: 10.1038/ncomms7004, PMID: 25633407 PMC4316748

[ref46] Fuhrmann D. Knoll L. J. Blakemore S. J. (2015). Adolescence as a sensitive period of brain development. Trends Cogn. Sci. 19, 558–566. doi: 10.1016/j.tics.2015.07.008 26419496

[ref47] Gautam P. Nuñez S. C. Narr K. L. Kan E. C. Sowell E. R. (2014). Effects of prenatal alcohol exposure on the development of white matter volume and change in executive function. Neuroimage Clin. 5, 19–27. doi: 10.1016/j.nicl.2014.05.010, PMID: 24918069 PMC4050317

[ref48] Girault J. B. Cornea E. Goldman B. D. Jha S. C. Murphy V. A. Li G.. (2020). Cortical structure and cognition in infants and toddlers. Cereb. Cortex 30, 786–800. doi: 10.1093/cercor/bhz126 31365070 PMC7306173

[ref49] Girault J. B. Cornea E. Goldman B. D. Knickmeyer R. C. Styner M. Gilmore J. H. (2019). White matter microstructural development and cognitive ability in the first 2 years of life. Hum. Brain Mapp. 40, 1195–1210. doi: 10.1002/hbm.24439 30353962 PMC6852619

[ref50] Guittard C. Novo A. Eutrope J. Gower C. Barbe C. Bednarek N.. (2023). Protocol for a prospective multicenter longitudinal randomized controlled trial (CALIN) of sensory-tonic stimulation to foster parent child interactions and social cognition in very premature infants. Front. Pediatr. 10:913396. doi: 10.3389/fped.2022.913396, PMID: 36727004 PMC9885178

[ref51] Guyer A. E. Pérez-Edgar K. Crone E. A. (2018). Opportunities for neurodevelopmental plasticity from infancy through early adulthood. Child Dev. 89, 687–697. doi: 10.1111/cdev.13073 29664997 PMC5948168

[ref52] Habibi A. Cahn B. R. Damasio A. Damasio H. (2016). Neural correlates of accelerated auditory processing in children engaged in music training. Dev. Cogn. Neurosci. 21, 1–14. doi: 10.1016/j.dcn.2016.04.003, PMID: 27490304 PMC6987702

[ref53] Hanson J. L. Nacewicz B. M. Sutterer M. J. Cayo A. A. Schaefer S. M. Rudolph K. D.. (2015). Behavioral problems after early life stress: contributions of the hippocampus and amygdala. Biol. Psychiatry 77, 314–323. doi: 10.1016/j.biopsych.2014.04.020, PMID: 24993057 PMC4241384

[ref54] Hardi F. A. Goetschius L. G. Peckins M. K. Brooks-Gunn J. McLanahan S. S. McLoyd V.. (2022). Differential developmental associations of material hardship exposure and adolescent amygdala-prefrontal cortex White matter connectivity. J. Cogn. Neurosci. 34, 1866–1891. doi: 10.1162/jocn_a_01801, PMID: 34942644 PMC9651170

[ref55] Huttenlocher P. R. de Courten C. (1987). The development of synapses in striate cortex of man. Hum. Neurobiol. 6, 1–9. PMID: 3583840

[ref56] Hyde K. L. Lerch J. Norton A. Forgeard M. Winner E. Evans A. C.. (2009). Musical training shapes structural brain development. J. Neurosci. 29, 3019–3025. doi: 10.1523/JNEUROSCI.5118-08.2009, PMID: 19279238 PMC2996392

[ref57] Ireland K. Iyer T. A. Penhune V. B. (2019). Contributions of age of start, cognitive abilities and practice to musical task performance in childhood. PLoS One 14:e0216119. doi: 10.1371/journal.pone.0216119, PMID: 31022272 PMC6483258

[ref58] Isbell E. Stevens C. Pakulak E. Hampton Wray A. Bell T. A. Neville H. J. (2017). Neuroplasticity of selective attention: research foundations and preliminary evidence for a gene by intervention interaction. Proc. Natl. Acad. Sci. U. S. A. 114, 9247–9254. doi: 10.1073/pnas.1707241114, PMID: 28819066 PMC5584441

[ref59] Jaramillo V. Volk C. Maric A. Furrer M. Fattinger S. Kurth S.. (2020). Characterization of overnight slow-wave slope changes across development in an age-, amplitude-, and region-dependent manner. Sleep 43:38. doi: 10.1093/sleep/zsaa038, PMID: 32154557

[ref60] Jeong H. J. Durham E. L. Moore T. M. Dupont R. M. McDowell M. Cardenas-Iniguez C.. (2021). The association between latent trauma and brain structure in children. Transl. Psychiatry 11:240. doi: 10.1038/s41398-021-01357-z, PMID: 33895776 PMC8068725

[ref61] Jernigan T. L. Trauner D. A. Hesselink J. R. Tallal P. A. (1991). Maturation of human cerebrum observed in vivo during adolescence. Brain 114, 2037–2049. doi: 10.1093/brain/114.5.2037 1933232

[ref62] Jolles D. Supekar K. Richardson J. Tenison C. Ashkenazi S. Rosenberg-Lee M.. (2016b). Reconfiguration of parietal circuits with cognitive tutoring in elementary school children. Cortex 83, 231–245. doi: 10.1016/j.cortex.2016.08.004, PMID: 27618765 PMC5160046

[ref63] Jolles D. Wassermann D. Chokhani R. Richardson J. Tenison C. Bammer R.. (2016a). Plasticity of left perisylvian white-matter tracts is associated with individual differences in math learning. Brain Struct. Funct. 221, 1337–1351. doi: 10.1007/s00429-014-0975-6, PMID: 25604464 PMC4819785

[ref64] Kanemaru N. Watanabe H. Taga G. (2012). Increasing selectivity of interlimb coordination during spontaneous movements in 2- to 4-month-old infants. Exp. Brain Res. 218, 49–61. doi: 10.1007/s00221-012-3001-3, PMID: 22249434

[ref65] Khundrakpam B. S. Reid A. Brauer J. Carbonell F. Lewis J. Ameis S.. (2013). Brain development cooperative group. Developmental changes in organization of structural brain networks. Cereb. Cortex 23:2072. doi: 10.1093/cercor/bhs187, PMID: 22784607 PMC3729193

[ref66] Kuhl U. Friederici A. D. LEGASCREEN consortium Skeide M. A. (2020). Early cortical surface plasticity relates to basic mathematical learning. NeuroImage 204:116235. doi: 10.1016/j.neuroimage.2019.116235 31586675

[ref67] Le Grand R. Mondloch C. J. Maurer D. Brent H. P. (2003). Expert face processing requires visual input to the right hemisphere during infancy. Nat. Neurosci. 6, 1108–1112. doi: 10.1038/nn1121, PMID: 12958600

[ref68] Lebel C. Mattson S. N. Riley E. P. Jones K. L. Adnams C. M. May P. A.. (2012). A longitudinal study of the long-term consequences of drinking during pregnancy: heavy in utero alcohol exposure disrupts the normal processes of brain development. J. Neurosci. 32, 15243–15251. doi: 10.1523/JNEUROSCI.1161-12.2012, PMID: 23115162 PMC3515671

[ref69] Lehto K. Mäestu J. Kiive E. Veidebaum T. Harro J. (2016). BDNF Val66Met genotype and neuroticism predict life stress: a longitudinal study from childhood to adulthood. Eur. Neuropsychopharmacol. 26, 562–569. doi: 10.1016/j.euroneuro.2015.12.029 26738427

[ref70] Mackes N. K. Mehta M. A. Beyh A. Nkrumah R. O. Golm D. Sarkar S.. (2022). A prospective study of the impact of severe childhood deprivation on brain White matter in adult adoptees: widespread localized reductions in volume but unaffected microstructural organization. eNeuro 9, ENEURO.0188–ENEU22.2022. doi: 10.1523/ENEURO.0188-22.2022, PMID: 36376082 PMC9665880

[ref71] Mareckova K. Miles A. Liao Z. Andryskova L. Brazdil M. Paus T.. (2022). Prenatal stress and its association with amygdala-related structural covariance patterns in youth. Neuroimage Clin. 34:102976. doi: 10.1016/j.nicl.2022.102976, PMID: 35316668 PMC8938327

[ref72] Marin M. M. (2009). Effects of early musical training on musical and linguistic syntactic abilities. Ann. N. Y. Acad. Sci. 1169, 187–190. doi: 10.1111/j.1749-6632.2009.04777.x, PMID: 19673778

[ref73] Martel M. Finos L. Koun E. Farnè A. Roy A. C. (2021). The long developmental trajectory of body representation plasticity following tool use. Sci. Rep. 11:559. doi: 10.1038/s41598-020-79476-8 33436755 PMC7804961

[ref74] Matejko A. A. Price G. R. Mazzocco M. M. Ansari D. (2013). Individual differences in left parietal white matter predict math scores on the preliminary scholastic aptitude test. NeuroImage 66, 604–610. doi: 10.1016/j.neuroimage.2012.10.045 23108272

[ref75] Matsudaira I. Yokota S. Hashimoto T. Takeuchi H. Asano K. Asano M.. (2016). Parental praise correlates with posterior insular cortex gray matter volume in children and adolescents. PLoS One 11:e0154220. doi: 10.1371/journal.pone.0154220, PMID: 27101139 PMC4839741

[ref76] Moore S. R. McEwen L. M. Quirt J. Morin A. Mah S. M. Barr R. G.. (2017). Epigenetic correlates of neonatal contact in humans. Dev. Psychopathol. 29, 1517–1538. doi: 10.1017/S0954579417001213, PMID: 29162165

[ref77] Moreno S. Bialystok E. Barac R. Schellenberg E. G. Cepeda N. J. Chau T. (2011). Short-term music training enhances verbal intelligence and executive function. Psychol. Sci. 22, 1425–1433. doi: 10.1177/0956797611416999, PMID: 21969312 PMC3449320

[ref78] Moreno S. Lee Y. Janus M. Bialystok E. (2015). Short-term second language and music training induces lasting functional brain changes in early childhood. Child Dev. 86, 394–406. doi: 10.1111/cdev.12297 25346534 PMC4376572

[ref79] Moreno S. Marques C. Santos A. Santos M. Castro S. L. Besson M. (2009). Musical training influences linguistic abilities in 8-year-old children: more evidence for brain plasticity. Cereb. Cortex 19, 712–723. doi: 10.1093/cercor/bhn120 18832336

[ref80] Murphy K. M. Beston B. R. Boley P. M. Jones D. G. (2005). Development of human visual cortex: a balance between excitatory and inhibitory plasticity mechanisms. Dev. Psychobiol. 46, 209–221. doi: 10.1002/dev.20053 15772972

[ref81] Nguyen T. V. Gower P. Albaugh M. D. Botteron K. N. Hudziak J. J. Fonov V. S.. (2016). The developmental relationship between DHEA and visual attention is mediated by structural plasticity of cortico-amygdalar networks. Psychoneuroendocrinology 70, 122–133. doi: 10.1016/j.psyneuen.2016.05.003, PMID: 27236606 PMC4907862

[ref82] Nguyen T. V. Wu M. Lew J. Albaugh M. D. Botteron K. N. Hudziak J. J.. (2017). Dehydroepiandrosterone impacts working memory by shaping cortico-hippocampal structural covariance during development. Psychoneuroendocrinology 86, 110–121. doi: 10.1016/j.psyneuen.2017.09.013, PMID: 28946055 PMC5659912

[ref83] Noble K. G. Houston S. M. Kan E. Sowell E. R. (2012). Neural correlates of socioeconomic status in the developing human brain. Dev. Sci. 15, 516–527. doi: 10.1111/j.1467-7687.2012.01147.x, PMID: 22709401 PMC6554027

[ref84] Ouss L. Le Normand M. T. Bailly K. Leitgel Gille M. Gosme C. Simas R.. (2018). Developmental trajectories of hand movements in typical infants and those at risk of developmental disorders: an observational study of kinematics during the first year of life. Front. Psychol. 9:83. doi: 10.3389/fpsyg.2018.00083, PMID: 29515472 PMC5826068

[ref85] Palomar-García M. Á. Hernández M. Olcina G. Adrián-Ventura J. Costumero V. Miró-Padilla A.. (2020). Auditory and frontal anatomic correlates of pitch discrimination in musicians, non-musicians, and children without musical training. Brain Struct. Funct. 225, 2735–2744. doi: 10.1007/s00429-020-02151-1, PMID: 33029708

[ref86] Paredes M. F. James D. Gil-Perotin S. Kim H. Cotter J. A. Ng C.. (2016). Extensive migration of young neurons into the infant human frontal lobe. Science 354:AAF7073. doi: 10.1126/science.aaf7073, PMID: 27846470 PMC5436574

[ref87] Patel Y. Parker N. Shin J. Howard D.. (2021). Virtual histology of cortical thickness and shared neurobiology in 6 psychiatric disorders. JAMA Psychiatry 78, 47–63. doi: 10.1001/jamapsychiatry.2020.2694 32857118 PMC7450410

[ref88] Penfield W. Boldrey E. (1937). Somatic motor and sensory representation in the cerebral cortex of man as studied by electrical stimulation. Brain 60, 389–443. doi: 10.1093/brain/60.4.389

[ref89] Penfield W Jasper H. (1954) Epilepsy and the functional anatomy of the human brain. Oxford, England: Little, Brown & Co, 47, 704

[ref90] Penhune V. Watanabe D. Savion-Lemieux T. (2005). The effect of early musical training on adult motor performance: evidence for a sensitive period in motor learning. Ann. N. Y. Acad. Sci. 1060, 265–268. doi: 10.1196/annals.1360.049, PMID: 16597774

[ref91] Piccolo L. R. Noble K. G. Pediatric Imaging, Neurocognition, and Genetics Study (2018). Perceived stress is associated with smaller hippocampal volume in adolescence. Psychophysiology 55:e13025. doi: 10.1111/psyp.13025, PMID: 29053191 PMC5899620

[ref92] Pisch M. Wiesemann F. Karmiloff-Smith A. (2019). Infant wake after sleep onset serves as a marker for different trajectories in cognitive development. J. Child Psychol. Psychiatry 60, 189–198. doi: 10.1111/jcpp.12948, PMID: 29989661

[ref93] Price A. J. Collado-Torres L. Ivanov N. A. Xia W. Burke E. E. Shin J. H.. (2019). Divergent neuronal DNA methylation patterns across human cortical development reveal critical periods and a unique role of CpH methylation. Genome Biol. 20:196. doi: 10.1186/s13059-019-1805-1, PMID: 31554518 PMC6761727

[ref94] Provenzi L. Grumi S. Altieri L. Bensi G. Bertazzoli E. Biasucci G.. (2021). Prenatal maternal stress during the COVID-19 pandemic and infant regulatory capacity at 3 months: a longitudinal study. Dev. Psychopathol. 35, 35–43. doi: 10.1017/S0954579421000766 34210369

[ref95] Pugin F. Metz A. J. Wolf M. Achermann P. Jenni O. G. Huber R. (2015). Local increase of sleep slow wave activity after three weeks of working memory training in children and adolescents. Sleep 38, 607–614. doi: 10.5665/sleep.4580, PMID: 25669190 PMC4355900

[ref96] Puhlmann L. M. Derome M. Morosan L. Kilicel D. Vrtička P. Debbané M. (2023). Longitudinal associations between self-reported attachment dimensions and neurostructural development from adolescence to early adulthood. Attach Hum. Dev. 25, 162–180. doi: 10.1080/14616734.2021.1993628, PMID: 34730475

[ref97] Qiu A. Zhang H. Wang C. Chong Y. S. Shek L. P. Gluckman P. D.. (2021). Canonical TGF-β signaling regulates the relationship between prenatal maternal depression and amygdala development in early life. Transl. Psychiatr. 11:170. doi: 10.1038/s41398-021-01292-z PMC7961018 33723212

[ref98] Remer J. Croteau-Chonka E. Dean D. C. D’Arpino S. Dirks H. Whiley D.. (2017). Quantifying cortical development in typically developing toddlers and young children, 1–6 years of age. NeuroImage 153, 246–261. doi: 10.1016/j.neuroimage.2017.04.010, PMID: 28392489 PMC5460988

[ref99] Rueda M. R. Posner M. I. Rothbart M. K. (2005). The development of executive attention: contributions to the emergence of self-regulation. Dev. Neuropsychol. 28, 573–594. doi: 10.1207/s15326942dn2802_2, PMID: 16144428

[ref100] Rusterholz T. Hamann C. Markovic A. Schmidt S. J. Achermann P. Tarokh L. (2018). Nature and nurture: brain region-specific inheritance of sleep neurophysiology in adolescence. J. Neurosci. 38, 9275–9285. doi: 10.1523/JNEUROSCI.0945-18.2018, PMID: 30249805 PMC6705989

[ref101] Sa de Almeida J. Lordier L. Zollinger B. Kunz N. Bastiani M. Gui L.. (2020). Music enhances structural maturation of emotional processing neural pathways in very preterm infants. NeuroImage 207:116391. doi: 10.1016/j.neuroimage.2019.116391, PMID: 31765804

[ref102] Saarikivi K. Putkinen V. Tervaniemi M. Huotilainen M. (2016). Cognitive flexibility modulates maturation and music-training-related changes in neural sound discrimination. Eur. J. Neurosci. 44, 1815–1825. doi: 10.1111/ejn.13176, PMID: 26797826

[ref103] Savion-Lemieux T. Bailey J. A. Penhune V. B. (2009). Developmental contributions to motor sequence learning. Exp. Brain Res. 195, 293–306. doi: 10.1007/s00221-009-1786-5 19363605

[ref104] Schlaug G. Norton A. Overy K. Winner E. (2005). Effects of music training on the child's brain and cognitive development. Ann. N. Y. Acad. Sci. 1060, 219–230. doi: 10.1196/annals.1360.015 16597769

[ref105] Schnack H. G. van Haren N. E. Brouwer R. M. Evans A. Durston S. Boomsma D. I.. (2015). Changes in thickness and surface area of the human cortex and their relationship with intelligence. Cereb. Cortex 25, 1608–1617. doi: 10.1093/cercor/bht357, PMID: 24408955

[ref106] Schoch S. F. Riedner B. A. Deoni S. C. Huber R. LeBourgeois M. K. Kurth S. (2018). Across-night dynamics in traveling sleep slow waves throughout childhood. Sleep 41:165. doi: 10.1093/sleep/zsy165, PMID: 30169809 PMC6231526

[ref107] Senju A. Vernetti A. Ganea N. Hudry K. Tucker L. Charman T.. (2015). Early social experience affects the development of eye gaze processing. Curr. Biol. 25, 3086–3091. doi: 10.1016/j.cub.2015.10.019, PMID: 26752077 PMC4683081

[ref108] Shin J. French L. Xu T. Leonard G. Perron M. Pike G. B.. (2018). Cell-specific gene-expression profiles and cortical thickness in the human brain. Cereb. Cortex 28, 3267–3277. doi: 10.1093/cercor/bhx197, PMID: 28968835 PMC13017599

[ref109] Sorrells S. F. Paredes M. F. Velmeshev D. Herranz-Pérez V. Sandoval K. Mayer S.. (2019). Immature excitatory neurons develop during adolescence in the human amygdala. Nat. Commun. 10:2748. doi: 10.1038/s41467-019-10765-1, PMID: 31227709 PMC6588589

[ref110] Steele C. J. Bailey J. A. Zatorre R. J. Penhune V. B. (2013). Early musical training and white-matter plasticity in the corpus callosum: evidence for a sensitive period. J. Neurosci. 33, 1282–1290. doi: 10.1523/JNEUROSCI.3578-12.2013, PMID: 23325263 PMC6704889

[ref111] Sterner K. N. Weckle A. Chugani H. T. Tarca A. L. Sherwood C. C. Hof P. R.. (2012). Dynamic gene expression in the human cerebral cortex distinguishes children from adults. PLoS One 7:e37714. doi: 10.1371/journal.pone.0037714, PMID: 22666384 PMC3364291

[ref112] Tanti A. Belliveau C. Nagy C. Maitra M. Denux F. Perlman K.. (2022). Child abuse associates with increased recruitment of perineuronal nets in the ventromedial prefrontal cortex: a possible implication of oligodendrocyte progenitor cells. Mol. Psychiatry 27, 1552–1561. doi: 10.1038/s41380-021-01372-y, PMID: 34799691 PMC9095471

[ref113] Traub F. Boynton-Jarrett R. (2017). Modifiable resilience factors to childhood adversity for clinical pediatric practice. Pediatrics 139:e20162569. doi: 10.1542/peds.2016-2569, PMID: 28557726

[ref114] Tröndle M. Popov T. Dziemian S. Langer N. (2022). Decomposing the role of alpha oscillations during brain maturation. elife 11:e77571. doi: 10.7554/eLife.77571, PMID: 36006005 PMC9410707

[ref115] Urbain C. De Tiège X. Op De Beeck M. Bourguignon M. Wens V. Verheulpen D.. (2016). Sleep in children triggers rapid reorganization of memory-related brain processes. NeuroImage 134, 213–222. doi: 10.1016/j.neuroimage.2016.03.055, PMID: 27039143

[ref116] Vandewouw M. M. Hunt B. A. E. Ziolkowski J. Taylor M. J. (2021). The developing relations between networks of cortical myelin and neurophysiological connectivity. NeuroImage 237:118142. doi: 10.1016/j.neuroimage.2021.118142, PMID: 33951516

[ref117] Vaquero L. Rousseau P. N. Vozian D. Klein D. Penhune V. (2020). What you learn & when you learn it: impact of early bilingual & music experience on the structural characteristics of auditory-motor pathways. NeuroImage 213:116689. doi: 10.1016/j.neuroimage.2020.116689 32119984

[ref118] Volk C. Jaramillo V. Studler M. Furrer M. O’Gorman Tuura R. L. Huber R. (2019). Diurnal changes in human brain glutamate + glutamine levels in the course of development and their relationship to sleep. NeuroImage 196, 269–275. doi: 10.1016/j.neuroimage.2019.04.040, PMID: 30991127

[ref119] Walther M. Berweck S. Schessl J. Linder-Lucht M. Fietzek U. M. Glocker F. X.. (2009). Maturation of inhibitory and excitatory motor cortex pathways in children. Brain Dev 31, 562–567. doi: 10.1016/j.braindev.2009.02.007, PMID: 19329268

[ref120] Wang C. Weng J. Yao Y. Dong S. Liu Y. Chen F. (2017). Effect of abacus training on executive function development and underlying neural correlates in Chinese children. Hum. Brain Mapp. 38, 5234–5249. doi: 10.1002/hbm.23728, PMID: 28727223 PMC6867117

[ref121] Weder N. Zhang H. Jensen K. Yang B. Z. Simen A. Jackowski A.. (2014). Child abuse, depression, and methylation in genes involved with stress, neural plasticity, and brain circuitry. J. Am. Acad. Child Adolesc. Psychiatry 53:417. doi: 10.1016/j.jaac.2013.12.025, PMID: 24655651 PMC4126411

[ref122] Westerhausen R. Luders E. Specht K. Ofte S. H. Toga A. W. Thompson P. M.. (2011). Structural and functional reorganization of the corpus callosum between the age of 6 and 8 years. Cereb. Cortex 21, 1012–1017. doi: 10.1093/cercor/bhq165, PMID: 20847151 PMC3077426

[ref123] Whittle S. Simmons J. G. Hendriksma S. Vijayakumar N. Byrne M. L. Dennison M.. (2016). Childhood maltreatment, psychopathology, and the development of hippocampal subregions during adolescence. Brain Behav. 7:e00607. doi: 10.1002/brb3.607, PMID: 28239518 PMC5318361

[ref124] Wijeakumar S. Kumar A. Delgado Reyes L. M. Tiwari M. Spencer J. P. (2019). Early adversity in rural India impacts the brain networks underlying visual working memory. Dev. Sci. 22:e12822. doi: 10.1111/desc.12822, PMID: 30803122 PMC6767418

[ref125] Wilhelm I. Kurth S. Ringli M. Mouthon A. L. Buchmann A. Geiger A.. (2014). Sleep slow-wave activity reveals developmental changes in experience-dependent plasticity. J. Neurosci. 34, 12568–12575. doi: 10.1523/JNEUROSCI.0962-14.2014 25209294 PMC6615503

[ref126] Williams P. G. Lerner M. A. (2019). COUNCIL ON EARLY CHILDHOOD; COUNCIL ON SCHOOL HEALTH. School readiness. Pediatrics 144:e20191766. doi: 10.1542/peds.2019-1766 31331984

[ref127] Wrigglesworth J. Ryan J. Vijayakumar N. Whittle S. (2019). Brain-derived neurotrophic factor DNA methylation mediates the association between neighborhood disadvantage and adolescent brain structure. Psychiatry Res. Neuroimaging 285, 51–57. doi: 10.1016/j.pscychresns.2018.12.012, PMID: 30771753

[ref128] Yeung M. S. Zdunek S. Bergmann O. Bernard S. Salehpour M. Alkass K.. (2014). Dynamics of oligodendrocyte generation and myelination in the human brain. Cells 159, 766–774. doi: 10.1016/j.cell.2014.10.011, PMID: 25417154

[ref129] Zacharopoulos G. Sella F. Cohen Kadosh K. Hartwright C. Emir U. Cohen K. R. (2021). Predicting learning and achievement using GABA and glutamate concentrations in human development. PLoS Biol. 19:e3001325. doi: 10.1371/journal.pbio.3001325, PMID: 34292934 PMC8297926

[ref130] Zhang X. Widaman K. Belsky J. (2021). Beyond orchids and dandelions: susceptibility to environmental influences is not bimodal. Dev. Psychopathol. 35, 191–203. doi: 10.1017/S0954579421000821, PMID: 34711295

[ref131] Zhou H. Yao Y. Geng F. Chen F. Hu Y. (2022). Right fusiform gray matter volume in children with long-term abacus training positively correlates with arithmetic ability. Neuroscience 507, 28–35. doi: 10.1016/j.neuroscience.2022.11.006, PMID: 36400323

[ref132] Zuk J. Vanderauwera J. Turesky T. Yu X. Gaab N. (2022). Neurobiological predispositions for musicality: white matter in infancy predicts school-age music aptitude. Dev. Sci. 26:e13365. doi: 10.1111/desc.13365, PMID: 36571291 PMC10291011

